# Developmental changes in effects of risk and valence on adolescent decision-making^☆^

**DOI:** 10.1016/j.cogdev.2013.04.001

**Published:** 2013-07

**Authors:** Laura K. Wolf, Nicholas D. Wright, Emma J. Kilford, Raymond J. Dolan, Sarah-Jayne Blakemore

**Affiliations:** a UCL Institute of Cognitive Neuroscience, 17 Queen Square, London WC1N 3AR, UK; b Wellcome Trust Centre for Neuroimaging, UCL,, 12 Queen Square, London WC1N 3BG, UK

**Keywords:** Risk-taking, Loss aversion, Valence, Decision-making, Adolescence

## Abstract

Recent research on risky decision-making in adults has shown that both the risk in potential outcomes and their valence (i.e., whether those outcomes involve gains or losses) exert dissociable influences on decisions. We hypothesised that the influences of these two crucial decision variables (risk and valence) on decision-making would vary developmentally during adolescence. We adapted a risk-taking paradigm that provides precise metrics for the impacts of risk and valence. Decision-making in 11–16 year old female adolescents was influenced by both risk and valence. However, their influences assumed different developmental patterns: the impact of valence diminished with age, while there was no developmental change in the impact of risk. These different developmental patterns provide further evidence that risk and valence are fundamentally dissociable constructs and have different influences on decisions across adolescence.

## Highlights

• Risk and valence influence choices in decision-making tasks.
• Adolescents aged 11–16 took part in a gambling task.
• Influences of risk and valence on decisions showed different development patterns.
• Risk-aversion remained stable while the influence of valence reduced with age.

## 1 Introduction

Value based decision-making involves an agent choosing from several alternatives based on the subjective values of available options. Two powerful influences on such decisions are risk in potential outcomes (Harrison & Rutstroem, 2008; Kacelnik & Bateson, 1996) and valence, meaning whether those outcomes involve gains or losses (Dayan & Seymour, 2008; Kahneman & Tversky, 1979). Risk can be defined as a state in which the decision-maker lacks precise knowledge about which outcome will follow from a decision – there is uncertainty. Individuals may be risk-averse (preferring lower risk options when comparing options with identical expected value (EV)), risk-neutral, or risk-seeking (preferring higher to lower risk options). Valence is defined as whether potential outcomes entail punishment (e.g., financial losses or painful electric shocks) or rewards (e.g., financial gains or tasty foods). The goal of the present study was to investigate the development of responses to these two crucial decision variables, risk and valence, from early- to mid-adolescence (aged 11–16 years).

### 1.1 The effect of valence on decisions in adolescence

We were particularly interested in the development of the impact of valence on decision-making, prompted in part by a recent study that investigated valence-dependent reversal learning in response to unexpected reward and punishment in adolescence (Van der Schaaf, Warmerdam, Crone, & Cools, 2011). Younger adolescents (age 10–11) displayed better reversal learning scores following a punishment than following a reward, and this difference in performance decreased with age across adolescence (from age 10 to 16). We investigated whether there is a similar development in the effect of valence on risky decision-making in early to mid-adolescence.

### 1.2 Development of risk-taking in adolescence

Previous studies have suggested that different aspects of risky decision-making show different developmental patterns (Blakemore & Robbins, 2012). Developmental trajectories of risk-taking behaviour differ depending on whether decisions are made in an affective or “hot” experimental context (e.g., when emotions are involved or peers are present) or a non-affective or “cold” context. In affective contexts, there is evidence that risk-taking peaks in mid-adolescence. A peak in reward-sensitivity in mid- to late adolescence (14–21 years) was found on a modified version of the Iowa Gambling Task (IGT; Cauffman et al., 2010). Participants in the IGT choose among four packs of cards, each associated with different profiles of monetary gain and loss (Bechara, Damasio, Damasio, & Anderson, 1994). Two packs are apparently lucrative but eventually result in significant loss (disadvantageous packs). The other two packs are “steady earners,” with small wins hardly ever penalised by even smaller losses (advantageous packs). Adults tend to sample the disadvantageous packs initially but then settle on the advantageous options. Cauffman and colleagues (2010) designed a modified version of the IGT in which gambling decisions were made about a particular deck on each trial, which enabled assessment of decision-making in response to gains or losses. There appeared to be a linear, age-related increase in the tendency to avoid the disadvantageous packs over the course of the task. However, compared with younger adolescents and adults, mid- to late adolescents learned more quickly to play from the advantageous packs, suggesting that this age group shows a heightened sensitivity to approaching rewards (Cauffman et al., 2010).

In a study employing a gambling task designed to induce relief or regret (Burnett, Bault, Coricelli, & Blakemore, 2010), a quadratic relationship emerged between age (9–35 years) and risk-taking, which peaked in mid-adolescence (around age 14). In a further study, adolescents (age 14–19) and adults (age 20+) played a card game in which cards could be turned over as long as gains were encountered, but as soon as participants received a loss the trial terminated (Figner, Mackinlay, Wilkening, & Weber, 2009a). Compared with adults, adolescents exhibited sub-optimal decision-making, failing to consider value and probability information when making decisions in an affective but not a non-affective version of the task. In a follow-up experiment, 10-year olds performed at a level similar to adults, suggesting that risk-taking in affective contexts peaks in adolescence but does not change in a non-affective context (Figner, Mackinlay, Wilkening, & Weber, 2009b).

In other gambling tasks where feedback is given but the context is non-affective, there is no evidence of a mid-adolescent peak in risk-taking; instead these tasks show a gradual decrease in risk-taking or no developmental change (Paulsen, Platt, Huettel, & Brannon, 2011; Rakow & Rahim, 2010; Van Leijenhorst et al., 2010). In a non-affective task in which participants aged 8–18 chose between a sure outcome and a gamble option (either high- or low-risk), risk-taking decreased across adolescence. Older adolescents chose low-risk gambles more frequently than high-risk gambles, and this difference was smaller in younger participants (Crone, Bullens, Van Der Plas, Kijkuit, & Zelazo, 2008). These studies suggest that risk-taking peaks in mid-adolescence in an affective context, while risk-taking remains stable or decreases in a non-affective context. However, it must be noted that the distinction between affective versus non-affective contexts is not always straightforward, which could lead to inconsistencies in the literature, especially because different studies employ different paradigms, age ranges, sample sizes, and measures of risk-taking.

While the affective context of decision-making tasks has been modulated in previous developmental studies, one aspect of decision-making that has not yet been examined is the differential impact of valence and risk. We use a non-affective task to isolate the effects of risk and valence on decisions (and developmental change in those effects), without studying how they interact with emotion.

### 1.3 Independent effects of valence and risk on decision-making in adults

The proposal that the impacts of risk and valence might show different developmental patterns is predicated on recent research suggesting that risk and valence have independent effects on adult decision-making (Wright et al., 2012). The prevailing view in psychology and economics has been that risk and valence are related in a specific fashion, with risk-aversion occurring for gains and risk-seeking for losses, given medium to high probabilities for both gain and loss outcomes (Kahneman & Tversky, 1979). An alternative hypothesis is that valence and risk exert independent influences on decisions in gambling tasks (Wright et al., 2012). This alternative hypothesis was motivated by evidence that multiple, interacting neural valuation systems influence decisions (Dayan, 2008), with processing of risk and valence by distinct neural systems being consistent with independent, rather than linked, behavioural effects. Behavioural and neurobiological evidence for a dissociation between the influences of risk and valence on decisions has been derived from studies that employed a financial gambling task that separately manipulated risk and valence (Wright et al., 2012).

### 1.4 The present study

We adapted the financial gambling task used by Wright et al. (2012) to obtain precise metrics for developmental changes in the impacts of risk and valence during adolescence (age 11–16). We first investigated whether adolescent decision-making is influenced by both risk and valence and next examined whether these influences are independent of one another. We then asked whether the influences of risk and valence on decision-making show different developmental patterns during adolescence. Developmental change was possible in the impact of either risk or valence, or of both, on decisions in this non-affective risk-taking task.

## 2 Method

### 2.1 Participants

Sixty-four female adolescents (mean age 13.9 years, range 11–16 years) took part. Data from three participants were excluded (two were unable to complete the task and one confused the buttons). All were recruited from the same academically selective secondary school in North London and were well matched for educational background and socioeconomic status. Participants were individually tested in a quiet classroom at their school.

Verbal IQ was assessed with the BPVS II (Dunn, Dunn, Whetton, & Burley, 1997) for all but seven participants (whose verbal IQ could not be assessed due to time limitations at the school). Verbal IQ was not associated with age (mean = 114.28, SD = 13.38, range 82–138; *β* = 0.002, *r*^2^ < 0.001, *p* > 0.9) and covarying verbal IQ did not affect any of our experimental results. Due to sex differences in brain maturation (Giedd et al., 1999), we tested only female participants.

### 2.2 Pretest of stimulus understanding

We first employed a validity check to ensure that participants understood basic information displayed in pie chart stimuli. Participants saw eight printed pairs of pie charts, and for each pair were told they should try to win as many points as possible (in the gain trials) or lose as few points as possible (in the loss trials) by choosing one of the two pie charts. We tested understanding of gains and losses (four pairs of pie charts contained only gains and four only losses), magnitudes (in four pairs, magnitudes differed while probabilities were identical between the two pie charts), and probabilities (magnitudes were identical and probabilities differed). Previous work has shown that 5-year olds understand simple probabilities (Schlottmann, 2001), and 8-year olds understand how risk and reward outcome contribute to a gamble's EV (Van Leijenhorst, Westenberg, & Crone, 2008). As expected, all participants met our inclusion criterion of over 75% accuracy (mean correct responses = 96.7%, SD = 6.4%).

### 2.3 The gambling task

**Fig. 1:**
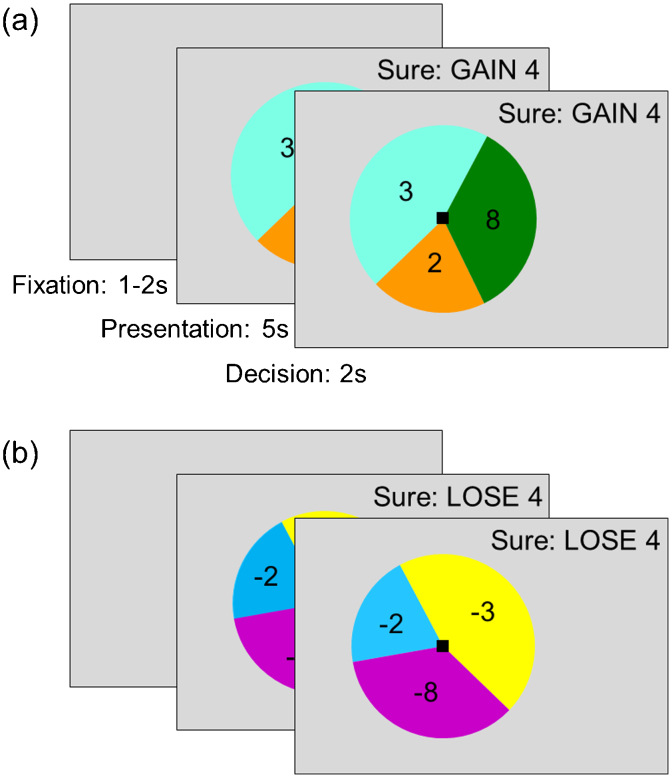
Experimental design. In each trial, participants were instructed to choose between a lottery and sure option. The lottery was represented by a pie chart with three segments corresponding to the three possible outcomes, with the size of each segment corresponding to the probability of that outcome occurring. The sure option was indicated on the upper right side of the screen. Half the trials involved winning points (“gain” trials) and half involved losing points (“loss” trials). (a) In each gain trial, participants chose either to accept a lottery (three varying possible outcomes, all ≥0) or reject it in favour of a sure gain of four points. (b) In each loss trial, participants chose either to accept a lottery (three varying possible outcomes, all ≤0) or reject it in favour of a sure loss of four points.

Participants completed a computer-based financial gambling task based on the “accept/reject” task used by Wright et al. (2012). In the “accept/reject” task used here (Fig. 1), participants performed 112 trials presented in random order, of which 56 were gain trials (all possible outcomes ≥0), and 56 were loss trials (all outcomes ≤0). In each trial, participants chose to accept either a lottery (with three possible outcomes) or a sure outcome (a gain of four points in gain trials, and a loss of four in loss trials). Each trial began with a fixation cross presented for 1–2 s (mean = 1.5 s), followed by a display of the options for 5 s. Finally, a black square appeared to signal that participants had 2 s to indicate their decision by pressing a button. Participants were informed that not responding resulted in the worst possible outcome, corresponding to zero points in the gain trials and loss of eight points in the loss trials. The 112 trials were split into two blocks of 56 trials, with each block lasting approximately 8 min. No feedback was given.

We manipulated risk by using a set of 56 lotteries (three possible outcomes, all ≥0), in which we parametrically and orthogonally manipulated degree of risk (i.e. variance; eight levels) and EV (seven levels). In adapting the paradigm for a younger age group it was not possible to fully control for skewness (a further aspect of risk), which ranged between −40 and 40 in the current sample. Half the lotteries had an EV above the sure amount, and half were below it. We presented each lottery in this set once to produce 56 gain trials. To manipulate valence, we multiplied all outcome amounts by −1 to produce 56 loss trials (i.e., all outcomes ≤0, and a sure option of −4). This created a set of gain trials and a set of matched loss trials.

Participants were told to treat the points as currency, at an exchange rate of one point equal to 50 pence. Participants began the testing session with an endowment of eight points. After the experiment, one gain trial and one loss trial were picked at random, and their outcomes were added to this endowment to determine a final payment. Participants could receive 0–16 points, which at the end of the task were converted into GBP (i.e., £0–8). Participants also received £1 for participation.

### 2.4 Stimulus sets

We generated a set of 56 lotteries by orthogonally manipulating the variance (8 levels; mean = 7.5, range 0.9–14.4) and EV (7 levels; mean = 4.0; range = 2.2–5.8) of the lottery. We created this stimulus set in two stages. First, we generated a list of every possible trial within the following constraints: each lottery had three outcomes (three pie chart segments); outcomes were between 0 and 8 points; the smallest allowable probability was 0.1 (to avoid probability distortion); and the smallest allowable probability increment was 0.05. Next, we selected the 56 trials that most closely matched our desired eight levels of variance and seven levels of EV.

### 2.5 Data analysis

Expected value of half the lotteries was above the sure amount and half below (mean EV across all 56 trials was equal to the sure option in both gain and loss trials). Thus, the proportion of riskier decisions was used as a metric of participants’ *risk preference* (PropRisk: risk-neutral = 0.5; risk-averse < 0.5; risk-seeking > 0.5). To derive an individual measure for the effect of valence on decisions, the *impact of valence* was calculated as the difference in proportion of riskier decisions between gain and loss trials (ImpValence = PropRisk_gain_ − PropRisk_loss_). Wright et al. (2012) used the same metrics for risk and valence.

We used these participant-derived parameters in two separate analyses. First, we assessed whether adolescent decision-making is influenced by both risk and valence. To examine whether risk influenced decisions, we performed a one-sample t-test to assess whether overall PropRisk (PropRiskall i.e., collapsed across gains and losses) was significantly different from 0.5. To examine whether valence influenced decisions, we performed a one-sample t-test to assess whether ImpValence was significantly different from 0. Second, we used regression analyses to determine whether these influences on decision-making are independent and whether they show different developmental patterns during adolescence. To examine whether the effect of age on valence was distinct from that of age on risk, we performed a forced entry multiple regression with age and PropRisk_all_ (proportion of riskier decisions collapsed across gain and loss trials) as predictors and ImpValence (the difference in proportion of riskier decisions between gain and loss trials) as the dependent variable.

## 3 Results

### 3.1 Task performance

Participants performed the task well, with non-response rates (total 2.1 ± 2.8%, gains 1.6 ± 2.9%, losses 2.6 ± 3.2%) similar to levels previously reported in adults (Wright et al., 2012), and not associated with age (*β* = 0.007, *r*^2^ < 0.001, *p* > 0.9).

### 3.2 Risk and valence influence decisions

**Fig. 2:**
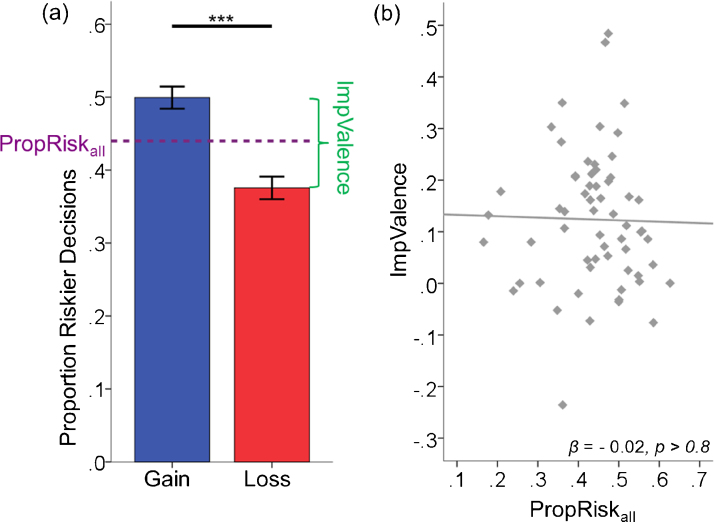
Risk and valence both influenced decisions, and individuals’ preferences for both were not associated (a) Individuals were significantly risk-averse overall (PropRisk_all_ < 0.5) as opposed to risk-neutral. Valence (ImpValence = PropRisk_gain_ − PropRisk_loss_) also significantly influenced decisions, with more gambling for gains than losses. (b) Individuals’ preferences related to risk (PropRisk_all_) and valence (ImpValence) were not associated. Error bars indicate standard error. ***p < 0.001.

We first examined whether both risk and valence influence decisions. Half the lotteries had an EV above the sure amount and half below (mean EV across all 56 trials was equal to the sure option with both gains and losses), which provided a simple metric of risk preference for each participant indexed as the proportion of riskier decisions made (PropRisk; risk-neutral = 0.5; risk-averse < 0.5; risk-seeking > 0.5). Individuals were, on average, significantly averse to risk (PropRisk_all_ = 0.44 ± 0.10) as opposed to risk-neutral, *t*_(60)_ = −4.9, *p* < 0.001 (Fig. 2a).

We also extracted a simple metric for the impact of valence for each participant from the difference in riskier decisions between gain and loss trials (ImpValence = PropRisk_gain_ − PropRisk_loss_). Individuals were also sensitive to valence (ImpValence = 0.12 ± 0.13), *t*_(60)_ = 7.3, *p* < 0.001 (Fig. 2a). Individuals selected the riskier option more frequently in gain trials than in loss trials (PropRisk_gain_ = 0.50 ± 0.12; PropRisk_loss_ = 0.38 ± 0.12), *t*_(60)_ = 7.3, *p* < 0.001. Participants were risk-neutral with gains, *t*_(60)_ = −0.04, *p* > 0.9, and risk-averse with losses, *t*_(60)_ = −8.0, *p* < 0.001. In sum, both risk and valence influence decisions, as has previously been shown with adults (Wright et al., 2012).

We next asked whether risk and valence had independent impacts on decision-making using regression analysis. Consistent with previous adult data (Wright et al., 2012), the impacts of risk and valence on individuals’ decisions were not associated with each other, *β* = −0.02, *r*^2^ < 0.001, *p* > 0.8 (Fig. 2b).

### 3.3 Developmental changes in the influences of risk and valence on decisions

**Fig. 3:**
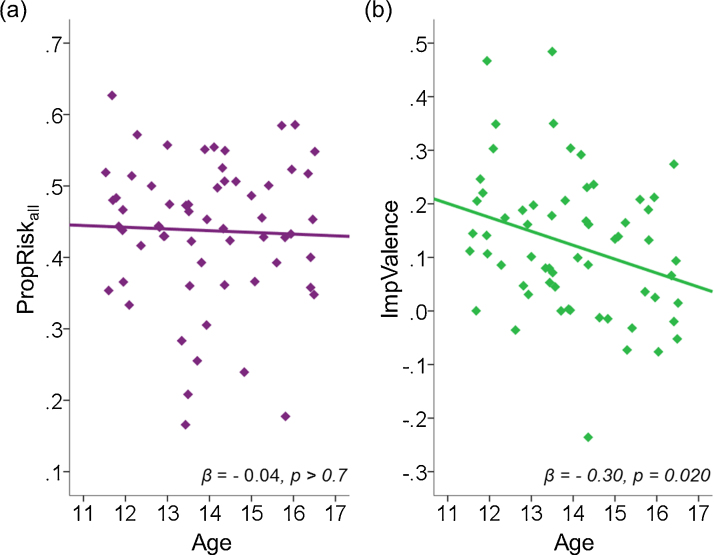
The impacts of risk and valence have different developmental patterns. We asked how the impacts of risk (PropRisk_all_) and valence (ImpValence) on decisions changed with age. (a) The influence of risk overall (PropRisk_all_) did not change with age. (b) The impact of valence (ImpValence) decreased significantly with age.

Finally, we asked how the impacts of risk and valence on decisions changed with age. Our data revealed that the influences of risk and valence show different developmental patterns (Fig. 3). The influence of risk on decisions did not change with age, *β* = −0.04, *r*^2^ = 0.001, *p* > 0.7 (Fig. 3a). In contrast, the impact of valence on decision-making decreased with age, *β* = −0.30, *r*^2^ = 0.09, *p* = 0.02 (Fig. 3b).

To demonstrate that the effect of age on valence was distinct from the effect of age on risk, we performed a forced entry multiple regression with age and PropRisk_all_ as predictors and ImpValence as the dependent variable. Age, as a single independent variable, significantly predicted ImpValence, *β* = −0.30, *r*^2^ = 0.089, *p* = 0.02. When PropRisk_all_ was added as a second independent variable, the effect of age on ImpValence remained significant, *β* = −0.30, *p* = 0.02. PropRisk_all_ did not predict ImpValence, *β* = −0.03, Δ*r*^2^ = 0.001, *p* > 0.8. The change in the impact of valence during adolescence was not driven by a change in responses to either gains or losses alone, with neither PropRisk_gain_, *β* = −0.20, *r*^2^ = 0.04, *p* = 0.13, nor PropRisk_loss_, *β* = 0.13, *r*^2^ = 0.02, *p* > 0.3, significantly predicted by age.

## 4 Discussion

We have demonstrated that risk and valence influence decision-making in 11–16-year-old female adolescents. The degrees to which risk and valence impact individuals’ decisions are not related, consistent with previous data for adults (Wright et al., 2012). Moreover, the influences of risk and valence show different developmental patterns across the age range studied: While the impact of risk did not change with age, the degree to which valence influenced decisions diminished with age (Fig. 3).

### 4.1 Risk-taking in adolescence is stable in this non-affective task

Previous work has suggested that the development of risky decision-making in affective and non-affective tasks varies during adolescence. Several studies have reported a peak in risk-taking in mid-adolescence for affective tasks, such as those involving emotions (Burnett et al., 2010; Cauffman et al., 2010; Figner et al., 2009a; Figner et al., 2009b), whereas non-affective tasks demonstrate either no change or a decrease in risk-taking with age (Crone et al., 2008; Figner et al., 2009a; Figner et al., 2009b; Paulsen et al., 2011; Rakow and Rahim, 2010). In the present study, we adapted a non-affective task used by Wright et al. (2012) to dissociate the influences of risk and valence on decision-making in adults. Adolescents were risk-averse in this gambling task, meaning that they made fewer riskier than safe decisions. We also demonstrated distinct developmental patterns in the impacts of risk and valence during early to mid-adolescence.

Developmental stability in risk-taking between early and mid-adolescence has been reported in previous studies examining risky decision-making in a non-affective task (Figner et al., 2009a). In contrast, tasks employing an affective context often find evidence for a peak in risk-taking around mid-adolescence (Burnett et al., 2010; Figner et al., 2009a). Our results support those by Figner et al., suggesting that, in the absence of affective task components, the propensity to take risks does not change during adolescence.

### 4.2 The impact of valence declines across early to mid-adolescence

Adolescent decisions were influenced by valence, such that fewer riskier decisions were made for losses than for gains. The effect of valence on risky decision-making decreased across adolescence: Compared with mid-adolescents, younger adolescents were more biased away from the riskier option by losses relative to gains. This effect was not driven by a change in responses to gains or losses alone; thus, the decrease in the effect of valence could be explained by a symmetrical reduction of the difference between risky decisions in the gain and loss domains. This would suggest that the effect does not derive from younger adolescents more frequently choosing the computationally simpler option in each trial. In a previous study requiring adolescents to learn to select advantageous decks of cards and to avoid selecting disadvantageous decks, there was an age-related increase in the propensity to avoid the disadvantageous decks over the course of the experiment (Cauffman et al., 2010). Older participants learned more quickly to avoid playing the disadvantageous decks; this was interpreted as an increase in loss-aversion during adolescence. In contrast, the reduction in the impact of valence during adolescence seen here is consistent with a recent study examining changes during adolescence in the effect of valence during a probabilistic reversal learning decision task (Van der Schaaf et al., 2011). In that study the effect of valence on decisions declined with increasing age (from 10 to 16 years), such that younger adolescents displayed greater sensitivity to unexpected financial losses compared to unexpected gains than did older adolescents. Thus, the developmental change in the effect of valence seen in our gambling task might reflect more general developmental changes in the influence of such approach-avoidance processes on decisions (Van der Schaaf et al., 2011).

### 4.3 Distinct developmental patterns of the impacts of risk and valence

Our finding provides a new source of evidence in a debate between models of risky economic decision-making. The prevailing view in psychology and economics is that risk and valence are related to each other in a specific fashion (risk-aversion for gains and risk-seeking for losses for the probabilities used in the current task), and that these preferences arise as the product of a utility function concave for gains and convex for losses (Kahneman and Tversky, 1979; Tversky and Kahneman, 1992). An alternative neurobiology-based hypothesis is that valence and risk exert independent influences on economic decisions (Wright et al., 2012). This hypothesis is supported by behavioural evidence for a dissociation between the influences of risk and valence on adults’ decisions, and by neural evidence for dissociable neural substrates related to the manipulations of valence and risk in orbitofrontal and parietal cortices, respectively (Wright et al., 2012). Our finding that the effects of risk and valence show different developmental patterns during adolescence is readily accommodated by neurobiological models where multiple, interacting neural decision-systems contribute to decision-making (Dayan, 2008). For example, decision-systems themselves (or those regulating the balance between them) might develop differently across adolescence. Further, these neurobiological models expand upon existing risk-return models of risky decisions (Bossaerts, 2010; Markowitz, 1952), which do not include effects of valence. We found adolescent participants were more risk-averse for losses than gains, which parallels the findings by Wright et al. (2012) and contrasts with other studies, which often report that participants are risk-seeking in the loss domain and risk-averse in the gain domain, for the probabilities used in this study (Kahneman and Tversky, 1979). This difference could be due to the format of the tasks. For each trial in the present study, participants considered whether to accept a lottery or reject it in favour of a sure option; when the lottery contains losses this could induce participants to avoid it. When instead individuals were asked to evaluate and select one of two options, they could not express avoidance by withdrawal but could potentially avoid losses by selecting the riskier option. Such a variant of our task used with adults (Wright et al., 2012) showed precisely this reversal in the direction of the valence effect (i.e., more gambling for gains than for losses). With respect to Prospect Theory (Kahneman and Tversky, 1979), while our data do not support the ‘reflection effect’ (i.e., risk-seeking with losses and risk-aversion with gains), an approach-avoidance account is consistent with ‘loss aversion’ where losses have greater weight (‘loom larger’) than gains.

### 4.4 Limitations and implications

As noted earlier, changes to the task format can change the direction of the effect of valence. Future developmental studies could examine the effects of valence and risk on decisions in other paradigms and other domains of risky decision-making – for example, in mixed gain-loss gambles. This study used simple metrics of the proportion of risky decisions made. Future work could also dissociate the developmental trajectories of responses to specific components of risk, such as variance and skewness, which have both been shown to influence adults’ decisions (Symmonds et al., 2011).

Despite the developmental change in the influence of valence over adolescence, collapsing across age, adolescents show similar impacts of risk and valence as found in adults (Wright et al., 2012). Therefore, adolescents and adults might generally use similar strategies when making risky decisions in a non-affective context. One explanation for the gradual decrease in the influence of valence during adolescence is a developmental shift in cognitive strategies used to make decisions in the kind of task employed here.

The previous study with adults employed two versions of the task. In one, for each trial there was a fixed evaluation period before the decision period (as in the present study). In the other, individuals were free to respond at any point the stimuli were displayed. There was no difference in the effects of risk or valence across these two versions of the task for adults (Wright et al., 2012), but we have not examined whether this is true for adolescents.

We intentionally avoided including an affective component in our task, so that we could study the effects of risk and valence on decisions without the influence of emotion. Future studies could examine how an affective context (for example, the presence of peers) interacts with the influence of risk and valence on decisions.

In sum, we show that the impacts of risk and valence on decision-making in a non-affective context show different developmental patterns. While the influence of risk does not change with age, the impact of valence decreases during adolescence. We speculate that these results may have implications for public health policy. Types of public health information provided, and the way information is presented, should be tailored to specific age groups to maximise impact. Parsing the influences on risky decisions and understanding how they develop will help determine which to stress for a given age group. Framing something as a loss, for example, may be more effective with younger than older adolescents.

## References

[bib0005] Bechara A., Damasio A.R., Damasio H., Anderson S.W. (1994). Insensitivity to future consequences following damage to human prefrontal cortex. Cognition.

[bib0010] Blakemore S.-J., Robbins T.W. (2012). Decision-making in the adolescent brain. Nature Neuroscience.

[bib0015] Bossaerts P. (2010). Risk and risk prediction error signals in anterior insula. Brain Structure and Function.

[bib0020] Burnett S., Bault N., Coricelli G., Blakemore S.-J. (2010). Adolescents’ heightened risk-seeking in a probabilistic gambling task. Cognitive Development.

[bib0025] Cauffman E., Shulman E.P., Steinberg L., Claus E., Banich M.T., Graham S. (2010). Age differences in affective decision making as indexed by performance on the Iowa Gambling Task. Developmental Psychology.

[bib0030] Crone E.A., Bullens L., Van Der Plas E.a.A., Kijkuit E.J., Zelazo P.D. (2008). Developmental changes and individual differences in risk and perspective taking in adolescence. Development and Psychopathology.

[bib0035] Dayan P. (2008). The role of value systems in decision making. Better than conscious? Decision making, the human mind and implications for institutions.

[bib0040] Dayan P., Seymour B. (2008). Values and actions in aversion. Neuroeconomics: Decision making and the brain.

[bib0045] Dunn L.M., Dunn L.M., Whetton C., Burley J. (1997). British Picture Vocabulary Scale.

[bib0050] Figner B., Mackinlay R.J., Wilkening F., Weber E.U. (2009). Affective and deliberative processes in risky choice: Age differences in risk taking in the Columbia Card Task. Journal of Experimental Psychology: Learning, Memory and Cognition.

[bib0055] Figner B., Mackinlay R.J., Wilkening F., Weber E.U. (2009). Risky choice in children, adolescents, and adults: Affective versus deliberative processes and the role of executive functions. Proceedings of the Society for Research in Child Development.

[bib0060] Giedd J.N., Blumenthal J., Jeffries N.O., Castellanos F.X., Liu H., Zijdenbos A. (1999). Brain development during childhood and adolescence: A longitudinal MRI study. Nature Neuroscience.

[bib0065] Harrison G.W., Rutstroem E.E., Cox J.C., Harrison G.W. (2008). Risk aversion in the laboratory.

[bib0070] Kacelnik A., Bateson M. (1996). Risky theories: The effects of variance on foraging decisions. American Zoologist.

[bib0075] Kahneman D., Tversky A. (1979). Prospect theory: Analysis of decision under risk. Econometrica.

[bib0080] Markowitz H. (1952). Portofolio Selection.

[bib0085] Paulsen D.J., Platt M.L., Huettel S.A., Brannon E.M. (2011). Decision-making under risk in children, adolescents, and young adults. Frontiers in Psychology.

[bib0090] Rakow T., Rahim S.B. (2010). Developmental insights into experience-based decision making. Journal of Behavioral Decision Making.

[bib0095] Schlottmann A. (2001). Children's probability intuitions: Understanding the expected value of complex gambles. Child Development.

[bib0100] Symmonds M., Wright N.D., Bach D.R., Dolan R.J. (2011). Deconstructing risk: Separable encoding of variance and skewness in the brain. Neuroimage.

[bib0105] Tversky A., Kahneman D. (1992). Advances in prospect-theory: Cumulative representation of uncertainty. Journal of Risk and Uncertainty.

[bib0110] Van der Schaaf M.E., Warmerdam E., Crone E.A., Cools R. (2011). Distinct linear and non-linear trajectories of reward and punishment reversal learning during development: Relevance for dopamine's role in adolescent decision making. Developmental Cognitive Neuroscience.

[bib0115] Van Leijenhorst L., Moor B.G., De Macks Z.A.O., Rombouts S.A.R.B., Westenberg P.M., Crone E.A. (2010). Adolescent risky decision-making: Neurocognitive development of reward and control regions. Neuroimage.

[bib0120] Van Leijenhorst L., Westenberg P.M., Crone E.A. (2008). A developmental study of risky decisions on the Cake Gambling Task: Age and gender analyses of probability estimation and reward evaluation. Developmental Neuropsychology.

[bib0125] Wright N.D., Symmonds M., Hodgson K., Fitzgerald T.H.B., Crawford B., Dolan R.J. (2012). Approach-avoidance processes contribute to dissociable impacts of risk and loss on choice. Journal of Neuroscience.

